# Stage-dependent endoplasmic reticulum homeostasis in osteogenic differentiation

**DOI:** 10.3389/fcell.2026.1809292

**Published:** 2026-04-23

**Authors:** Jili Wang, Xiaoyu Li, Xiaoge Wang, Linyi Wang, Yandong Zhang, Xiaokun Dong, Xiangpeng Lu, Shangzeng Wang

**Affiliations:** 1 The Second Affiliated Hospital of Henan University of Chinese Medicine, Zhengzhou, China; 2 Henan Provincial Hospital of Traditional Chinese Medicine, Zhengzhou, China; 3 Henan University of Chinese Medicine, Zhengzhou, China; 4 Luohe Medical College, Luohe, China; 5 The first Affiliated Hospital of Henan University of Chinese Medicine, Zhengzhou, China

**Keywords:** calcium homeostasis, endoplasmic reticulum stress, ER-phagy, lipid metabolism, osteogenic differentiation

## Abstract

Osteoporosis (OP) is primarily characterized by reduced bone mass, microarchitectural deterioration, and an increased susceptibility to fragility fractures. A central pathological feature of OP is the progressive impairment of osteogenic differentiation and bone matrix production in osteoblasts. The endoplasmic reticulum (ER), a pivotal organelle responsible for secretory protein folding, lipid/sterol biosynthesis, and intracellular Ca^2+^ storage, is subjected to a substantial secretory burden during osteogenic differentiation and functions as a critical regulatory hub integrating metabolic stress, inflammatory signaling, and mineralization-associated calcium signaling. Emerging evidence indicates that disruption of ER homeostasis regulates osteogenic differentiation through the three canonical branches of the unfolded protein response (UPR), including PERK–eIF2α–ATF4, IRE1α–XBP1, and ATF6 signaling pathways. In parallel, selective ER autophagy (ER-phagy) dynamically regulates ER quality control during osteogenic differentiation through removal of damaged ER domains and misfolded substrates such as procollagen. In addition, ER Ca^2+^ stores and STIM/ORAI-mediated store-operated Ca^2+^ entry (SOCE) cooperatively maintain calcium homeostasis during osteogenesis and regulate spatiotemporal expression of osteogenic transcription factors, including Runx2 and Sp7, through Ca^2+^ oscillatory signaling. ER membrane lipid composition further modulate osteogenic fate by influencing membrane contact site dynamics and cellular metabolic adaptation. In this review, we systematically summarize the crosstalk among ER stress, ER-phagy, Ca^2+^ homeostasis, and lipid metabolism during osteogenic differentiation from the perspective of ER structure–function coupling. We further discuss potential therapeutic strategies targeting ER stress regulation, including chemical chaperones and UPR/autophagy modulators, to provide new insights for targeted therapeutic approaches for osteoporosis.

## 1 Introduction

Osteoporosis (OP) is a systemic skeletal disorder characterized by decreased bone mass, deterioration of bone microarchitecture, and an increased risk of fragility fractures (Ye et al., 2025; Morin et al., 2023). The onset and progression of OP are closely linked to disruption of the dynamic balance between bone formation and bone resorption. With the rapid aging of the global population, OP has emerged as a highly prevalent condition associated with a substantial disability burden (Salari et al., 2021). Fragility fractures represent the most severe clinical complication of OP. In 2019, the global incidence of fractures reached approximately 2296 per 10 million individuals and continues to rise, imposing significant burdens on public health systems and socioeconomic structures worldwide (GBD 2019 Fracture Collaborators, 2021). Therefore, elucidating the regulatory mechanisms underlying bone formation and identifying novel therapeutic targets are of critical importance for improving OP prevention and treatment strategies. Osteogenic differentiation is a continuous and tightly regulated biological process in which bone marrow mesenchymal stem cells (BMSCs) progressively differentiate into mature osteoblasts and ultimately acquire a mineralized phenotype (Ponzetti and Rucci, 2021; Komori, 2006). As osteoblasts advance toward the maturation and mineralization stages, they undergo a functional shift toward high-throughput synthesis and secretion of bone matrix proteins, including type I collagen and non-collagenous matrix components (Rutkovskiy et al., 2016; Schlesinger et al., 2020). This transition markedly increases the cellular demand for protein folding capacity, secretory pathway efficiency, and organelle homeostasis. Consequently, the endoplasmic reticulum (ER)–centered secretory and quality control network becomes a critical determinant of osteoblast functional integrity and continuity during osteogenic differentiation.

The ER is a central organelle responsible for secretory protein folding and processing, lipid biosynthesis, and intracellular Ca^2+^ storage, and its functional state is tightly coupled to cellular secretory demand. When protein folding demand exceeds ER processing capacity, endoplasmic reticulum stress (ER stress) is induced and activates the unfolded protein response (UPR), an adaptive signaling network that restores ER homeostasis or triggers maladaptive responses under persistent stress (Hetz and Papa, 2018; Hetz et al., 2020). In addition, the ER contributes to multi-layered intracellular homeostasis regulation through Ca^2+^ reservoir control, membrane lipid biogenesis, and membrane contact site networks, thereby playing an essential role in maintaining the functional stability of highly secretory cells (Crul and Maléth, 2021; Jacquemyn et al., 2017; Wenzel et al., 2022). Recent evidence indicates that ER dysfunction is closely associated with bone metabolic disorders (Huang et al., 2023; Iyer and Adams, 2023; Zhong et al., 2023). However, most existing studies focus on individual ER pathways or specific signaling axes, and systematic reviews that dynamically integrate ER stress, selective ER autophagy (ER-phagy), Ca^2+^ homeostasis regulation, and ER-associated lipid metabolism in the context of changing functional demands during osteogenic differentiation remain limited (Alekos et al., 2020; Guo J. et al., 2021). Furthermore, the functional outcome of endoplasmic reticulum (ER) stress is determined by both its duration and intensity. Transient or adaptive ER stress is characterized by a temporary increase in BiP/GRP78, XBP1s, or p-eIF2α/ATF4, without sustained CHOP induction or impairment of osteogenic function. In contrast, persistent ER stress leads to sustained activation of UPR signaling, accompanied by continuous upregulation of CHOP, GADD34, and cleaved caspase-3, as well as functional impairments, including ER Ca^2+^ dyshomeostasis, decreased ALP activity, and reduced mineralization capacity (Huang et al., 2023; Turishcheva et al., 2022; Wu et al., 2023; Saito et al., 2011). Accordingly, ER homeostasis can be operationally defined as an “adaptive range” and a “maladaptive (decompensated) range.” The adaptive range is characterized by transient activation of BiP/GRP78, XBP1s, and p-eIF2α/ATF4 without sustained CHOP induction, indicating that ER function remains within a compensatory regulatory capacity. In contrast, sustained elevation of CHOP, GADD34, and cleaved caspase-3, together with impaired osteogenic function, indicates that ER stress has exceeded the homeostatic threshold (Huang et al., 2023; Turishcheva et al., 2022; Wu et al., 2023). In this review, we systematically summarize the coordinated regulatory roles of ER stress, ER-phagy, Ca^2+^ homeostasis, and ER-related lipid metabolism during osteogenic differentiation from the perspective of dynamic functional demand adaptation. Furthermore, we discuss potential therapeutic strategies centered on restoration of ER homeostasis, aiming to provide an integrated framework for OP mechanism research and bone regeneration therapy.

## 2 Literature search strategy

To ensure the comprehensiveness and relevance of this review, a systematic literature search was conducted using the PubMed database, covering the period from database inception to February 2026.

During the search process, terms related to osteogenic differentiation and bone metabolism (including “osteogenesis,” “osteogenic differentiation,” “osteoblast,” “bone formation,” and “osteoporosis”) were used in combination with ER-related functional terms (such as “endoplasmic reticulum stress,” “ER stress,” “unfolded protein response,” “UPR,” “PERK,” “IRE1,” and “ATF6”), as well as ER-associated regulatory mechanisms (including “ER-phagy,” “calcium homeostasis,” “store-operated calcium entry,” “SOCE,” “STIM,” “ORAI,” and “lipid metabolism”). These terms were combined using Boolean operators (AND/OR) to identify relevant studies on ER homeostasis regulation and osteogenic differentiation.

Eligible studies were prioritized if they focused on ER-related mechanisms (including ER stress/UPR, ER-phagy, calcium homeostasis, and lipid metabolism) and were relevant to osteogenic differentiation, osteoblast function, or osteoporosis. Particular emphasis was placed on *in vitro* and *in vivo* studies with clearly defined mechanistic insights, while representative review articles were included to provide contextual background and conceptual support. Studies with limited relevance or lacking mechanistic evidence were excluded. In addition, backward reference screening of the included studies was performed to ensure comprehensive coverage of key literature.

## 3 Structure and function of the endoplasmic reticulum

The ER is a key organelle responsible for maintaining protein folding and secretory processing in highly secretory cells, and its structural state directly determines the capacity of cells to sustain secretory function under high secretory load conditions (Schwarz and Blower, 2016; Sun and Brodsky, 2019; Hetz et al., 2015). During osteogenic differentiation, as bone matrix protein synthesis and mineralization-related secretory demands progressively increase, osteoblasts gradually acquire a high-secretory functional phenotype. This transition imposes increased demand on ER structural capacity and stability, positioning the ER as a critical structural foundation for maintaining the continuity of osteogenic differentiation and mineralization function (Blair et al., 2017; Tohmo et al., 2011; Sohaskey et al., 2010). Therefore, defining ER structural characteristics and their relationship with secretory load adaptation is essential for understanding how osteoblasts maintain high secretory activity.

The ER consists of a continuous membranous network, including the nuclear envelope and the peripheral ER extending into the cytoplasm, forming a dynamic architecture composed of interwoven sheet-like and tubular structures (Westrate et al., 2015; Terasaki et al., 2013). This continuous membrane system exhibits high structural plasticity and undergoes morphological rearrangement and domain remodeling in response to cellular differentiation stages and functional demands (Schwarz and Blower, 2016). Under high secretory conditions, expansion of rough ER sheets and increased ribosome density represent hallmark structural adaptations (Schwarz and Blower, 2016; Sohaskey et al., 2010; Westrate et al., 2015; Nyathi et al., 2013). These changes enhance nascent polypeptide translocation, folding, and trafficking efficiency, thereby providing structural support for sustained synthesis and secretion of bone matrix proteins (Iyer and Adams, 2023; Lynes and Simmen, 2011).

**FIGURE 1:**
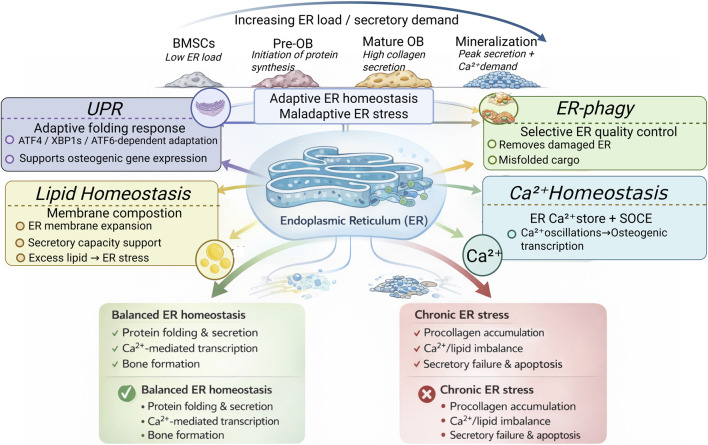
Stage-dependent regulation of endoplasmic reticulum homeostasis during osteogenic differentiation. As bone marrow mesenchymal stem cells (BMSCs) progressively differentiate into pre-osteoblasts (Pre-OB), mature osteoblasts (Mature OB), and finally enter the mineralization stage, ER load and secretory demand gradually increase. To accommodate these dynamic changes, the endoplasmic reticulum (ER) integrates multiple regulatory mechanisms, including the unfolded protein response (UPR), ER-phagy, Ca^2+^ homeostasis, and lipid homeostasis, to coordinate protein folding, ER membrane expansion, secretory capacity, and osteogenic transcriptional activity, thereby preserving adaptive ER homeostasis. This adaptive state supports efficient protein folding and secretion, Ca^2+^-dependent transcription, and bone matrix production, ultimately promoting bone formation. In contrast, when ER stress exceeds the buffering capacity of homeostatic regulation, maladaptive ER stress ensues, resulting in procollagen accumulation, Ca^2+^/lipid imbalance, secretory dysfunction, and apoptosis, which in turn impair osteogenic differentiation and mineralization. Overall, this schematic illustrates the dynamic changes in ER homeostasis across distinct stages of osteogenic differentiation and highlights its dual role in determining osteogenic outcomes.

In addition, the ER serves as a central hub for membrane lipid synthesis and Ca^2+^ storage, both of which depend on the integrity and continuity of the ER membrane network (Jacquemyn et al., 2017; Schwarz and Blower, 2016; Meldolesi and Pozzan, 1998). Disruption of membrane system integrity can compromise secretory pathway continuity and destabilize Ca^2+^-dependent protein folding processes (Preissler et al., 2020; Halbleib et al., 2017). During the late stages of osteogenic differentiation, insufficient membrane lipid supply or impaired Ca^2+^ reservoir homeostasis can disrupt ER structural stability, thereby reducing secretory load adaptability and suppressing matrix mineralization (Jacquemyn et al., 2017; Saito et al., 2011; Sriburi et al., 2004; Bommiasamy et al., 2009; Wu et al., 2014). Collectively, through its continuous and dynamic membrane network architecture, the ER provides the structural foundation for secretory pathway organization under high secretory load conditions in osteoblasts and supports secretory processing and Ca^2+^-dependent protein folding (Figure 1).

## 4 ER stress regulates osteogenic differentiation

**FIGURE 2:**
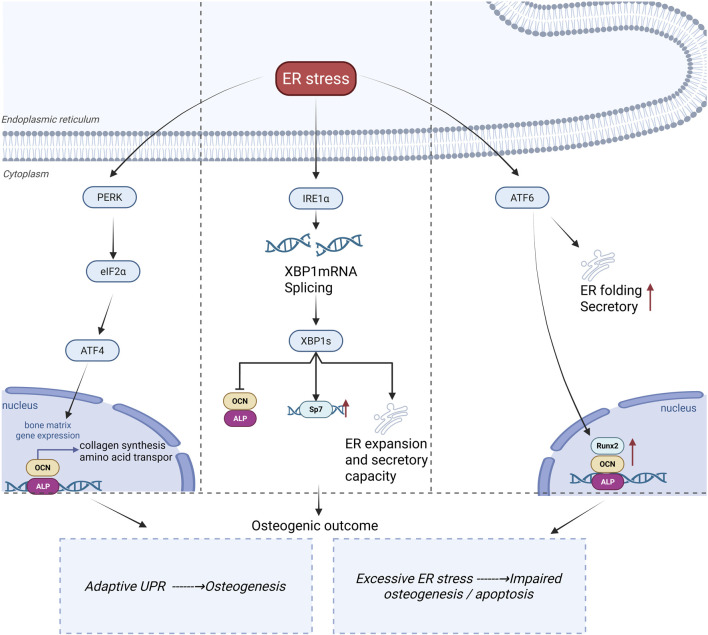
Canonical unfolded protein response signaling in the control of osteogenic differentiation during ER stress. ER stress triggers the three canonical branches of the unfolded protein response (UPR): PERK–eIF2α–ATF4, IRE1α–XBP1s, and ATF6. These pathways coordinate transcriptional and secretory adaptations that enable osteoblast-lineage cells to cope with increased protein-folding demand. PERK signaling promotes ATF4-dependent programs involved in bone matrix gene expression, collagen synthesis, and amino acid transport; IRE1α-dependent XBP1 mRNA splicing generates XBP1s, which supports osteogenic transcriptional activity and enhances ER expansion and secretory capacity; and ATF6 activation reinforces ER folding and secretory function while promoting the expression of osteogenic regulators, including Runx2, OCN, and ALP. Thus, adaptive UPR signaling is required to maintain ER homeostasis and sustain osteogenic differentiation, whereas excessive or chronic ER stress disrupts these processes, leading to impaired osteogenesis and apoptosis. This figure highlights the dual, context-dependent roles of canonical UPR signaling in determining osteogenic fate.

**FIGURE 3:**
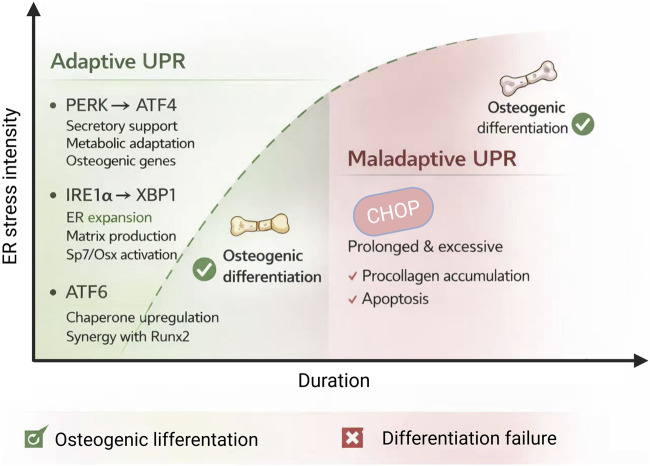
Intensity and duration dependent effects of UPR during osteogenic. This diagram summarizes the biphasic roles of the unfolded protein response (UPR) during osteogenic differentiation. Mild and transient UPR activation enhances ER folding capacity, promotes ER expansion, and supports adaptive secretory function, thereby facilitating osteogenic transcriptional programs. In contrast, prolonged or excessive UPR activation drives chronic ER stress, leading to apoptotic signaling and suppression of osteogenic differentiation. The functional outcome of UPR signaling is determined by its intensity, duration, and the specific stage of osteogenic differentiation.

During osteogenic differentiation, ER stress represents a homeostatic adaptive state characterized by threshold- and time-dependent features (Hetz et al., 2020; Helander et al., 2014; Hetz, 2012). Depending on stress intensity, duration, and differentiation stage, ER stress can drive divergent biological outcomes. Moderate and transient ER stress promotes adaptive enhancement of ER folding capacity and secretory fitness through activation of cytoprotective unfolded protein response (UPR) signaling, thereby supporting osteogenic differentiation (Jang et al., 2012). In contrast, chronic ER stress exceeding ER adaptive buffering capacity shifts UPR signaling from adaptive regulation toward pro-apoptotic and function-suppressive outputs, ultimately impairing osteogenic differentiation (Hetz et al., 2020; Hetz, 2012). UPR is mediated by the signaling network composed of PERK–eIF2α–ATF4, IRE1α–XBP1, and ATF6. The functional output of this network is determined by stress intensity, duration, and the stage of osteogenic differentiation (Hetz et al., 2020), as shown in Figures 2,3.

### 4.1 PERK–eIF2α–ATF4 axis and osteogenic differentiation

The PERK–eIF2α–ATF4 axis represents a central signaling module mediating protein folding load sensing and adaptive translation–transcription reprogramming during ER stress (Wang et al., 2018). Its primary function is to convert ER protein folding burden into an adaptive output integrating global translation attenuation and selective transcriptional activation (Teske et al., 2011; B'Chir et al., 2013). In BMP2-induced osteogenic differentiation models (including Perk-deficient mice and primary osteoblasts), as well as in PTH-treated osteoblast models (in combination with PERK/ATF4 inhibition or siRNA-mediated knockdown), activation of the PERK–eIF2α–ATF4 axis significantly regulates osteogenic gene expression (Saito et al., 2011; Zhang et al., 2019). Upon nuclear translocation, ATF4 directly controls the transcription of osteocalcin, alkaline phosphatase, and multiple bone matrix genes, thereby promoting osteogenic differentiation. Across multiple genetic and *in vitro* osteogenic models, ATF4 functions as a central transcriptional regulator linking translational control to downstream differentiation programs. In osteoblast-specific Nf1-deficient mouse models, as well as in MC3T3-E1 cells and primary osteoblasts, siRNA-based studies targeting ATF4-related regulators demonstrate that ATF4 modulates collagen synthesis, amino acid transport, and metabolic gene expression, thereby maintaining osteoblast functional homeostasis under conditions of high secretory demand (Elefteriou et al., 2006; Yu et al., 2009; Franceschi et al., 2007). In ER stress–induced mouse cortical neuron models (with ATF4 deletion and CHOP knockdown) and ethanol-treated human primary BMSC models (via ATF4/CHOP silencing), sustained ATF4 activation enhances CHOP-mediated pro-apoptotic signaling, accompanied by impaired osteogenic differentiation capacity and lineage shift (Galehdar et al., 2010; Chen et al., 2013). Collectively, these findings suggest that under stress conditions such as inflammation, oxidative stress, or metabolic dysfunction, sustained ATF4 activation preferentially drives pro-apoptotic signaling cascades, thereby suppressing osteogenic differentiation.

Further studies indicate that the PERK–eIF2α–ATF4 axis exhibits a clear dependence on both stress intensity and duration during osteogenic differentiation. Under moderate and transient endoplasmic reticulum (ER) stress, PERK-mediated phosphorylation of eIF2α selectively enhances ATF4 translation, thereby promoting osteogenic gene expression and regulating differentiation programs in BMP2- or PTH-induced models. In addition, ATF4 supports osteoblast secretory adaptation by facilitating amino acid uptake and collagen synthesis (Saito et al., 2011; Teske et al., 2011; Zhang et al., 2019; Elefteriou et al., 2006). In contrast, under persistent or excessive stress conditions, ATF4 preferentially drives CHOP-mediated pro-apoptotic signaling, which is associated with impaired osteogenesis and increased apoptosis in bone-relevant stress models (Chen et al., 2013). Accordingly, the functional outcome of the PERK–eIF2α–ATF4 axis is determined by whether ER stress is maintained within an adaptive regulatory range.

Currently, evidence supporting the role of the PERK–eIF2α–ATF4 axis in osteogenic differentiation is mainly derived from *in vitro* osteogenic induction models and selected genetic intervention studies, indicating that this pathway is activated during osteogenesis and contributes to osteogenic gene transcription. The role of ATF4 in osteogenesis is supported by relatively strong causal evidence. Notably, this pathway may exhibit context-dependent biphasic effects depending on stress intensity and duration, although its precise functional limits remain to be fully defined.

### 4.2 IRE1α–XBP1 axis and osteogenic differentiation

The IRE1α–XBP1 axis is distinguished in osteogenic differentiation by its capacity to transcriptionally drive ER biogenesis and enhance secretory capacity, thereby supporting the dual structural and functional requirements of highly secretory osteoblasts (Tohmo et al., 2011; Sriburi et al., 2004). *In vitro* overexpression studies demonstrate that upon IRE1α activation, its endoribonuclease (RNase) activity mediates unconventional splicing of XBP1 mRNA, generating the transcriptionally active spliced form of XBP1 (XBP1s). This process induces the expression of genes involved in endoplasmic reticulum biogenesis, membrane expansion, and enhanced secretory capacity (Sriburi et al., 2004). In BMP-2–induced osteogenic differentiation models (including *in vitro* osteogenic induction systems, IRE1α conditional knockout mice, and siRNA-mediated intervention), osteogenic differentiation is consistently accompanied by increased XBP1s levels. Conversely, inhibition of IRE1α or XBP1 significantly reduces alkaline phosphatase activity and suppresses mineralized nodule formation (Tohmo et al., 2011; Tohmonda et al., 2015; Tohmonda et al., 2013). Collectively, these findings support a functional role for the IRE1α–XBP1 axis in regulating osteogenic differentiation. Mechanistically, XBP1s directly binds to and activates the promoter region of the core osteogenic transcription factor Sp7, thereby coupling ER homeostasis regulation with osteogenic transcriptional programs (Tohmo et al., 2011).

In addition, the IRE1α–XBP1 axis promotes phospholipid biosynthesis and ER membrane expansion (Sriburi et al., 2004), providing essential metabolic and membrane biosynthetic support for maintaining structural integrity and secretory capacity of osteoblasts during high secretory demand stages (Tohmo et al., 2011; Lee et al., 2005).

Currently, evidence supporting the role of the IRE1α–XBP1 axis in osteogenic differentiation is primarily derived from BMP2-induced *in vitro* models and genetic intervention studies, indicating that this pathway exerts a well-supported pro-osteogenic effect. XBP1s regulates key osteogenic transcription factors, such as Sp7 (Osx), and is supported by relatively strong causal evidence.

However, existing studies are largely limited to specific induction models, and the role of the IRE1α–XBP1 axis under chronic stress conditions or across different stages of osteogenic differentiation remains insufficiently characterized.

### 4.3 ATF6 regulation of osteogenic differentiation

ATF6 is one of the three canonical branches of the unfolded protein response (UPR). It primarily functions by sensing ER protein-folding demand and inducing the transcription of molecular chaperones and protein-folding–related genes, thereby enhancing ER folding capacity and secretory function. In vitro cell models, ATF6 undergoes proteolytic processing in the Golgi apparatus, generating a transcriptionally active cytosolic fragment that translocates to the nucleus to regulate target gene expression (Ye et al., 2000; Shen et al., 2002; Karagöz et al., 2019). During BMP-2-induced osteogenic differentiation, mild ER stress promotes ATF6 processing and nuclear translocation, leading to increased expression of osteogenic marker genes such as OCN. Conversely, inhibition of ATF6 significantly attenuates BMP-2- or Runx2-mediated ALP activity, OCN transcription, and matrix mineralization, supporting a necessary regulatory role of ATF6 in BMP-2-mediated osteogenic signaling (Jang et al., 2012). Certain small molecules can also modulate osteogenic differentiation by regulating ATF6 activity. In vitro pharmacological models, including C3H10T1/2 osteoprogenitor cells and rat bone marrow–derived mesenchymal stem cells (rBMSCs), curcumin enhances ATF6 transcriptional activity and upregulates the expression of key osteogenic markers, including Runx2, alkaline phosphatase (ALP), and osteocalcin, thereby promoting osteogenic differentiation (Son et al., 2018; Gu et al., 2012). However, compared with the PERK–eIF2α–ATF4 and IRE1α–XBP1 branches, studies on ATF6 in osteogenic differentiation remain relatively limited (Jang et al., 2012; Son et al., 2018). Evidence supporting a pro-osteogenic role for ATF6 is primarily derived from *in vitro* cell-based studies and pharmacological intervention experiments, suggesting its involvement in secretory adaptation and extracellular matrix (ECM) protein synthesis. However, the role of ATF6 across different stages of osteogenesis, under diverse pathological microenvironments, and its coordinated interaction with pathways such as ER-phagy and Ca^2+^ homeostasis remain incompletely understood. Furthermore, current understanding of how ATF6 activation intensity and duration influence osteogenic outcomes remains incomplete. The functional boundary of ATF6 between physiological adaptive remodeling and pathological ER stress responses requires further clarification (Hetz et al., 2020; Sharma et al., 2019),which currently limits translational evaluation of ATF6 as a potential therapeutic osteogenic target.

## 5 ER-phagy and osteogenic differentiation

ER-phagy is a subtype of selective autophagy that functions as a selective ER quality control mechanism. Its primary biological role is to remove damaged or excess ER subdomains, thereby enabling ER structural renewal and maintaining ER proteostasis within an adaptive homeostatic range.

**FIGURE 4:**
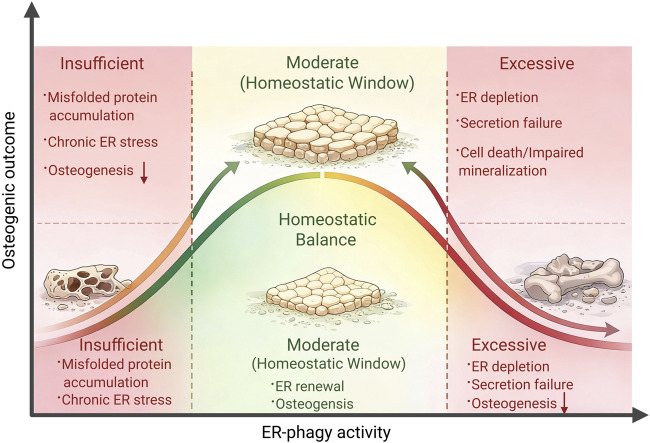
Stage-dependent roles of ER-phagy in maintaining ER homeostasis during osteogenic differentiation. In response to endoplasmic reticulum (ER) stress, cells engage ER-associated degradation (ERAD) and ER-phagy in concert to preserve ER homeostasis. ER-phagy primarily occurs through two distinct routes. In macro-ER-phagy, damaged or surplus ER fragments are selectively sequestered into autophagosomes and subsequently delivered for lysosomal degradation, a process mediated by ER-phagy receptors such as CCPG1 and TEX264 together with LC3-associated machinery. In micro-ER-phagy, ER fragments are directly engulfed and degraded by lysosome- or vacuole-like compartments. During osteogenic differentiation, ER-phagy displays a stage-dependent dynamic pattern. At the early stage, it mainly restrains the accumulation of misfolded proteins and helps maintain basal ER homeostasis. As cells enter the commitment stage and secretory demand rises, ER-phagy contributes to adaptation to the increasing protein-processing burden. During the maturation stage, when folding stress and abnormal cargo become more prominent, ER-phagy further facilitates the clearance of damaged ER and aberrant proteins. At the mineralization stage, ER-phagy is more closely associated with ER renewal and quality maintenance. Collectively, this schematic highlights the dual role of ER-phagy in stress adaptation and in supporting osteogenic differentiation across distinct developmental stages.

This process is distinct from macroautophagy, which mediates non-selective cytoplasmic degradation, and from ER-associated degradation (ERAD), which targets misfolded proteins for degradation via the ubiquitin–proteasome system. These pathways differ fundamentally in terms of substrate selectivity and degradation mechanisms. Based on these distinctions, ER-phagy can be further classified according to the mode of ER delivery to lysosomes, Based on the mode of ER delivery to lysosomes, ER-phagy can be broadly categorized into macro-ER-phagy and micro-ER-phagy (Chino and Mizushima, 2023). During macro-ER-phagy, ER-resident receptors (including members of the FAM134 family, CCPG1, TEX264, and others) interact with LC3/GABARAP through LC3-interacting regions (LIRs), recruiting ER fragments to the isolation membrane to form autophagosomes, which subsequently fuse with lysosomes for degradation. In contrast, micro-ER-phagy involves direct invagination of lysosomal or vacuolar membranes to engulf ER fragments, enabling ER structural remodeling and degradation of ER-associated proteins while alleviating ER proteotoxic burden (Chino and Mizushima, 2023; Ferro-Novick et al., 2021; Reggiori and Molinari, 2022). Under the persistent high secretory demand characteristic of osteogenic differentiation, ER-phagy does not primarily function as a direct driver of osteogenic lineage signaling. Instead, it acts as a structural proteostasis buffering mechanism that removes damaged or redundant ER regions, prevents chronic amplification of ER stress, and maintains ER function within a proteostasis range permissive for osteogenic differentiation. As shown in Figure 4.

### 5.1 Dynamic changes of ER-Phagy during osteogenic differentiation

Accumulating evidence indicates that ER-phagy is dynamically regulated during osteogenic differentiation in a stage-dependent manner according to secretory demand and proteostasis load (Cillo et al., 2025; Gui et al., 2025; De Leonibus et al., 2024). During the early stage of osteogenic commitment of BMSCs, the overall cellular secretory load remains relatively low. In cell-based models, gene perturbation studies (e.g., siRNA and CRISPR-mediated approaches) have demonstrated that receptor-mediated selective clearance of ER subdomains, such as that mediated by FAM134B, prevents the accumulation of aberrant ER structures (Forrester et al., 2019). At this stage, ER-phagy is maintained at a basal level, functioning to limit misfolded protein accumulation and preserve baseline ER homeostasis (Chino and Mizushima, 2023; Yang et al., 2021). This supports organelle stability during early differentiation and provides the structural foundation necessary for subsequent expansion of the secretory apparatus and enhancement of cellular function. Upon initiation of osteogenic commitment and progression into early differentiation, mTORC1–TFEB–mediated transcriptional regulation of autophagy plays a key regulatory role. Increased mTORC1 activity inhibits TFEB nuclear translocation, thereby transiently suppressing the transcription of autophagy-related genes. In parallel, the TFEB/TFE3–FAM134B axis has been shown to regulate ER-phagy at the transcriptional level in both cellular and tissue models (Martina et al., 2012; Cinque et al., 2020). Integrating these findings with evidence of dynamic mTORC1 activity changes in osteoprogenitor cells and *in vivo* osteogenic models (Chen and Long, 2015; Huang et al., 2015), ER-phagy flux may exhibit a stage-dependent reduction during early osteogenic differentiation under conditions of increasing secretory demand. During the proliferative stage, as osteoblasts sustain higher protein synthesis demand, ER-phagy flux is moderately upregulated. In this context, ER-phagy primarily functions as a proteostasis buffering mechanism that alleviates progressively accumulated folding stress while preventing structural ER depletion (Forrester et al., 2019; Smith et al., 2018; Liang et al., 2020; Liao et al., 2019). During osteoblast maturation, in collagen-secreting cells and misfolded procollagen models, selective ER-phagy mediated by the calnexin–FAM134B complex contributes to the quality control of endogenous procollagen. Inhibition of autophagy or knockdown of related genes results in increased accumulation of procollagen within the ER (Forrester et al., 2019; Ishida et al., 2009). These findings indicate that under conditions where type I collagen and other bone matrix proteins reach peak synthesis and secretion, ER-phagy plays a critical role in the clearance of misfolded proteins.

In this context, as secretory load markedly increases, ER-associated degradation (ERAD) alone becomes insufficient to handle the burden of misfolded protein clearance. Consequently, ER-phagy emerges as an important compensatory degradation pathway, contributing to the maintenance of secretory flux and ER homeostasis.

### 5.2 Insufficient ER-phagy leads to ER stress imbalance and osteogenic impairment

When ER-associated clearance capacity is compromised, osteoblasts exhibit reduced efficiency in removing damaged ER subdomains and folding-defective secretory precursors. As a result, abnormal protein load accumulates within the ER, leading to exacerbated ER stress and impaired secretory capacity (Li et al., 2018). Retention of misfolded procollagen and bone matrix proteins within the ER not only reduces secretion efficiency but also disrupts structural integrity of the bone matrix, ultimately resulting in impaired mineralization and reduced bone mass (Li et al., 2018; Nollet et al., 2014). In osteoblast-specific autophagy-deficient models, Atg7 deficiency markedly exacerbates ER stress and results in a significant reduction in bone mass, indicating a close association between impaired autophagy and osteogenic dysfunction. Importantly, Atg7 is a core autophagy gene, and its deletion reflects global macroautophagy impairment rather than a specific defect in ER-phagy. Therefore, these findings support a critical role for the autophagy–ER homeostasis axis in maintaining osteogenic function but cannot be considered direct evidence for the selective role of ER-phagy. Accordingly, studies based on Atg7 deficiency primarily reflect alterations in macroautophagy rather than receptor-mediated ER-phagy. In contrast, receptor-specific studies involving FAM134B, CCPG1, and TEX264 provide more direct support for the selective degradation mechanism of ER-phagy. Furthermore, pharmacological alleviation of ER stress using chemical chaperones significantly rescues autophagy deficiency–induced impairments in osteogenic differentiation, mineralization, and bone formation in both *in vitro* and *in vivo* models (Li et al., 2018; Nollet et al., 2014), thereby providing indirect evidence for the critical role of ER stress in this process.

### 5.3 The “dose–response” window and pathophysiological significance of ER-phagy

In osteogenesis-related pathological contexts, the functional outcome of ER-phagy is jointly determined by its activity level and duration, exhibiting a clear dose- and time-dependent pattern. In high glucose–induced osteoblast models, impairment of FAM134B-mediated ER-phagy exacerbates ER stress and suppresses osteogenesis (Gui et al., 2025). Similarly, in osteoblast-specific Atg7-deficient models, autophagy impairment leads to sustained ER stress activation and reduced bone mass (Li et al., 2018). These findings indicate that insufficient ER-phagy activity results in inadequate clearance of protein-folding stress and structural damage, thereby promoting a transition to chronic ER stress and impairing osteogenic phenotype maintenance. In addition, the MiT/TFE–FAM134B axis has been implicated in the adaptive transcriptional regulation of ER-phagy (Cinque et al., 2020). However, as this evidence is primarily derived from non-osteogenic cell models, its functional relevance in bone tissue remains to be further validated.

Conversely, when ER-phagy flux is moderately upregulated and primarily results in restoration of ER homeostasis, it can function as a compensatory quality control mechanism to alleviate proteotoxic stress and improve osteogenic phenotypes under pathological conditions such as metabolic dysfunction (Ferro-Novick et al., 2021; Gui et al., 2025; Smith and Wilkinson, 2017). However, sustained overactivation or dysregulated control of ER-phagy may also produce adverse outcomes. Previous studies have shown that excessive enhancement of FAM134B-mediated ER-phagy can accelerate ER degradation and is associated with persistent UPR activation and initiation of cell death programs (Liao et al., 2019; Mo et al., 2020). In non-osteogenic cell models, such as HeLa cells, excessive ER-phagy activity has been shown to induce ER structural depletion and sustained activation of ER stress (Liao et al., 2019). Based on evidence from bone-related review literature, similar mechanisms may impair the synthesis and secretion of bone matrix proteins by reducing ER secretory capacity; however, this hypothesis requires further validation in osteoblast-specific models (Yin et al., 2019). In addition, in osteoclast systems, excessive or dysregulated autophagy promotes osteoclast differentiation and exacerbates bone loss via the RANKL–NF-κB signaling pathway (Sun et al., 2021; DeSelm et al., 2011). Consistent with this, related reviews highlight the critical role of autophagy in osteoclast function (Montaseri et al., 2020), suggesting that imbalance in autophagy/ER-phagy may further amplify bone metabolic disorders through bone remodeling coupling mechanisms.

**FIGURE 5:**
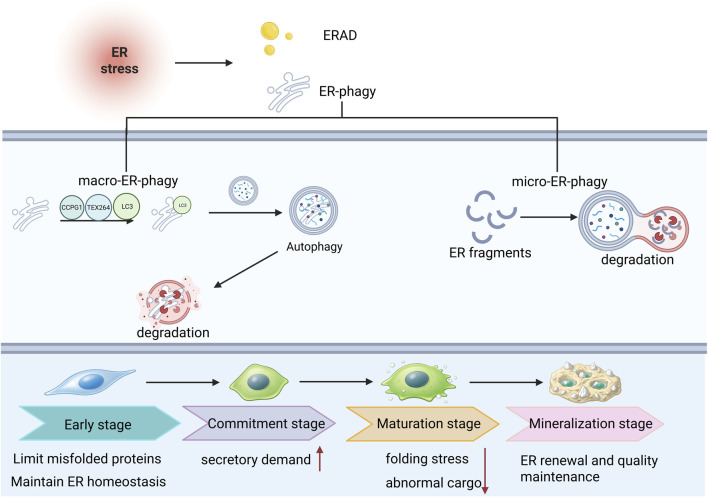
Optimal ER-phagy activity is required to sustainER homeostasis and osteogenic differentiation. This schematic illustrates the dose-dependent role of selective ER autophagy (ER-phagy) during osteogenic differentiation. Adequate ER-phagy removes damaged or excessive ER regions and misfolded secretory precursors, thereby preserving ER homeostasis and supporting osteogenic function under high secretory demand. Insufficient ER-phagy leads to ER stress accumulation and impaired osteogenesis, whereas excessive ER-phagy may cause ER depletion and reduced secretory capacity. Thus, ER-phagy must be maintained within an adaptive range to sustain ER homeostasis and promote osteogenic differentiation.

Collectively, current evidence indicates that ER-phagy exhibits a dose–response–dependent functional window during osteogenic differentiation: insufficient ER-phagy activity leads to persistent ER stress activation and impaired osteogenesis, whereas moderate activation maintains ER homeostasis and promotes osteogenic differentiation. In contrast, excessive ER-phagy may result in ER structural depletion and cellular damage. However, most available evidence is derived from ER-phagy receptor–related studies and autophagy-deficient models. Direct *in vivo* causal evidence targeting canonical ER-phagy receptors remains limited, and the precise functional boundaries of ER-phagy in osteoblasts have yet to be fully defined. As shown in Figure 5. Moderate ER-phagy maintains ER homeostasis and supports osteogenic differentiation.

## 6 ER Ca^2+^ homeostasis and osteogenic differentiation

During osteogenic differentiation, Ca^2+^ not only regulates osteogenesis-related gene expression but also supports ER proteostasis, secretory flux, and ER quality control network integrity.

### 6.1 ER as the major Ca^2+^ reservoir in osteoblast-lineage cells

During cellular differentiation, the folding, assembly, and maturation of type I collagen and multiple bone matrix proteins primarily occur within the ER lumen. Ca^2+^ is a critical determinant of molecular chaperone conformational stability and functional activity (e.g., calreticulin and calnexin). Stability of ER luminal Ca^2+^ concentration directly determines the efficiency of Ca^2+^-dependent protein folding reactions and thereby governs secretory proteostasis (Michalak et al., 2002; Ellgaard and Frickel, 2003; Carreras-Sureda et al., 2018). Depletion of ER Ca^2+^ stores disrupts Ca^2+^-dependent folding processes, leading to misfolded protein accumulation within the ER and activation of the unfolded protein response (UPR), ultimately compromising osteogenic differentiation capacity (Preissler et al., 2020; Wu et al., 2014; Mekahli et al., 2011). At the cellular level, ER Ca^2+^ depletion disrupts protein folding mediated by Ca^2+^-dependent molecular chaperones, such as BiP, leading to the accumulation of misfolded proteins and activation of the unfolded protein response (UPR) (Preissler et al., 2020). This mechanism is further supported by review literature, highlighting ER Ca^2+^ dyshomeostasis as a key upstream trigger of ER stress and UPR activation (Mekahli et al., 2011). In osteoblasts and *in vivo* models, disruption of ER Ca^2+^ homeostasis induces ER stress, suppresses osteogenic differentiation, and is associated with reduced bone mass (Wu et al., 2014). Moreover, sustained ER Ca^2+^ depletion drives a shift of the UPR from an adaptive response to a persistently activated state, thereby impairing cellular function (Pontisso et al., 2024). Collectively, these findings indicate that prolonged depletion of ER Ca^2+^ stores in osteoblasts may restrict secretory flux and inhibit osteogenic differentiation via sustained UPR activation.

### 6.2 STIM/ORAI-mediated store-operated Ca^2+^ entry

STIM/ORAI-mediated store-operated Ca^2+^ entry (SOCE) is a central mechanism for maintaining ER Ca^2+^ store homeostasis, primarily by enabling sustained refilling of ER luminal Ca^2+^ (Prakriya and Lewis, 2015). During osteogenic differentiation, store-operated Ca^2+^ entry (SOCE) maintains ER Ca^2+^ stores and plays a critical role in bone formation in both osteoblasts and animal models (Robinson et al., 2023). *In vitro*, Orai1-deficient osteoblasts (including MC3T3-E1 cells and primary osteoblasts) exhibit impaired Ca^2+^ influx, reduced alkaline phosphatase activity, and diminished mineralization capacity (Robinson et al., 2023; Hwang et al., 2012), indicating that SOCE is essential for maintaining the osteogenic phenotype. Mechanistically, Ca^2+^ within the ER lumen is essential for the function of Ca^2+^-dependent molecular chaperones, such as calreticulin and calnexin, which support protein folding and secretory function (Michalak et al., 2002). Impairment of SOCE compromises ER Ca^2+^ refilling, leading to sustained Ca^2+^ depletion, activation of the unfolded protein response (UPR), and induction of ER stress (Makio et al., 2024). In osteoblasts, persistent ER stress promotes apoptosis via CHOP-related pathways and suppresses osteogenic differentiation potential (Guo et al., 2014).

Moreover, severe disruption of Ca^2+^ homeostasis may become functionally coupled to ER turnover and clearance pathways, highlighting coordinated crosstalk among SOCE, UPR, and ER-phagy (Kwon et al., 2023; Song et al., 2018).

Under conditions of ER Ca^2+^ store depletion or impaired SOCE-mediated refilling, Ca^2+^-dependent molecular chaperones, such as calnexin and calreticulin, become functionally compromised, leading to the accumulation of misfolded proteins within the ER lumen. This subsequently activates canonical UPR pathways, including IRE1α, PERK, and ATF6, as indicated by XBP1 splicing and CHOP induction (Preissler et al., 2020; Michalak et al., 2002; Ellgaard and Frickel, 2003; Mekahli et al., 2011; Makio et al., 2024). UPR activation not only drives adaptive ER expansion through the transcriptional regulation of protein folding and degradation-related genes but also modulates ER-phagy via membrane remodeling and transcriptional control. This enhances the selective clearance of damaged or excess ER subdomains under high secretory load, thereby buffering folding stress and maintaining ER homeostasis (Kwon et al., 2023; Song et al., 2018).

In turn, ER-phagy-mediated ER membrane renewal and quality control influence SERCA-dependent Ca^2+^ reuptake and STIM1–ORAI1-mediated Ca^2+^ refilling by reshaping ER structural integrity and membrane contact site organization, thereby regulating ER Ca^2+^ storage capacity and Ca^2+^ dynamics (Makio et al., 2024; Gubas and Dikic, 2022; Burgoyne et al., 2015; Wu et al., 2018). Collectively, ER Ca^2+^ depletion, UPR activation, and ER remodeling/ER-phagy constitute a tightly coupled dynamic feedback network. By integrating protein folding capacity, secretory flux, and Ca^2+^ homeostasis, this network governs the activation and maintenance of key osteogenic transcriptional programs, including RUNX2 and SP7, ultimately determining the outcome of osteogenic differentiation.

### 6.3 Ca^2+^ oscillation patterns and osteogenic differentiation

The signaling function of Ca^2+^ during osteogenic differentiation is primarily mediated through spatiotemporal dynamics. Ca^2+^ oscillations function as a core calcium signaling code that integrates mechanical cues, biochemical signaling, and cellular homeostatic regulatory networks (Dolmetsch et al., 1998; Berridge et al., 2000; Uhlén and Fritz, 2010). Depletion of ER Ca^2+^ stores or insufficient store refilling typically results in reduced oscillation frequency or unstable Ca^2+^ signaling patterns, thereby weakening Ca^2+^-dependent downstream signaling pathways (e.g., CaM–CaMKII) and impairing precise temporal coordination of osteogenic transcriptional programs (Wakai et al., 2013; De Koninck and Schulman, 1998; Zayzafoon, 2006).

During osteogenic differentiation, mechanical loading, growth factor signaling (BMP/Wnt), and extracellular niche-derived cues induce Ca^2+^ oscillations with characteristic frequencies (0.1–10 mHz), which are closely associated with activation of osteogenic transcription factors such as Runx2 and Sp7 (Suzuki et al., 2013; Gao et al., 2023; Yu et al., 2017). In osteoblasts, Ca^2+^ oscillations have been shown to regulate downstream transcriptional programs via the Ca^2+^/CaM/CaMKII signaling pathway (Choi et al., 2013). CaMKII promotes the expression of osteogenic genes by modulating key transcription factors, such as Osterix, thereby linking Ca^2+^ dynamics to osteogenic transcriptional regulation. Disruption of ER Ca^2+^ stores or impaired refilling alters intracellular Ca^2+^ dynamics. In non-osteogenic models (e.g., mouse oocytes), defective ER Ca^2+^ refilling leads to reduced and unstable Ca^2+^ oscillations (Wakai et al., 2013), indicating that ER Ca^2+^ store integrity is essential for maintaining oscillatory stability. Collectively, ER Ca^2+^ dyshomeostasis may disrupt Ca^2+^ oscillatory patterns, attenuate CaM–CaMKII signaling, and impair the precise regulation of osteogenic transcriptional programs. Ca^2+^ oscillation stability is not solely determined by Ca^2+^ channel activity and store refilling systems but is also constrained by ER homeostatic status (Makio et al., 2024; Groenendyk et al., 2021). Sustained ER stress compromises secretory and protein folding capacity and disrupts key Ca^2+^ handling machinery, including SERCA and STIM1, thereby impairing ER Ca^2+^ store maintenance (Groenendyk et al., 2021; Carreras-Sureda et al., 2023). Furthermore, ER-phagy, as a major regulator of ER membrane remodeling and ultrastructural organization, when dysregulated, may alter ER membrane topology and spatial architecture. This can disrupt Ca^2+^ exchange at membrane contact sites and impair coordination of Ca^2+^ release and reuptake (Gubas and Dikic, 2022; Burgoyne et al., 2015; Wu et al., 2018).

Evidence for ER Ca^2+^ homeostasis regulation is primarily supported by Ca^2+^ functional assays and Orai1/STIM1-related genetic models. Depletion of ER Ca^2+^ stores has been shown to induce UPR activation and impair mineralization, providing relatively strong causal evidence. However, important limitations remain, as the quantitative relationships and stage-specific regulatory interactions among Ca^2+^ signaling, UPR, and ER-phagy are not yet well defined.

## 7 ER regulation of lipid metabolism and its relationship with osteogenic differentiation

During osteogenic differentiation, abnormal lipid composition reshapes ER membrane biophysical properties and induces lipid bilayer stress (LBS), leading to activation of the unfolded protein response and suppression of osteogenic differentiation. In parallel, the ER regulates metabolic reprogramming of cellular energetics and secretory load adaptation through coordinated lipid sensing–transcriptional regulatory networks, lipid droplet biogenesis, and membrane contact site-associated metabolic processes, collectively determining homeostatic maintenance and functional output during osteogenic differentiation (Halbleib et al., 2017; Volmer et al., 2013; Nohturfft et al., 1999; Nguyen et al., 2017; Talari et al., 2023).

### 7.1 Lipid homeostasis as a critical upstream regulator of ER stress–mediated osteogenic differentiation

During the maturation and mineralization stages of osteogenic differentiation, osteoblasts progressively establish a high-secretory phenotype characterized by sustained synthesis and secretion of type I collagen and other bone matrix proteins, placing increased demands on ER structural integrity and secretory proteostatic buffering capacity (Sohaskey et al., 2010). In this context, lipid homeostasis primarily influences osteogenic differentiation through ER membrane composition and topology sensing rather than through energy supply alone, thereby modulating ER adaptive capacity under conditions of high secretory demand.

Excess cholesterol accumulation or increased saturated fatty acid content alters ER membrane lipid composition and bilayer biophysical properties, disrupting the membrane microenvironment of transmembrane proteins and activating ER stress responses through lipid bilayer stress mechanisms. Notably, LBS can activate core UPR sensors (e.g., IRE1α and PERK) even in the absence of significant increases in misfolded protein load (Halbleib et al., 2017; Volmer et al., 2013).

Accumulating evidence indicates that cholesterol overload or saturated fatty acid exposure (e.g., palmitate) induces sustained UPR activation and suppresses osteogenic differentiation (Halbleib et al., 2017; Kitai et al., 2013; Volmer and Ron, 2015; Li K. et al., 2019; Yeh et al., 2014; Takeuchi et al., 2020). Under high secretory demand conditions, lipid overload–induced chronic ER stress impairs adaptive expansion of the secretory apparatus and lowers ER tolerance thresholds to folding and secretory stress, thereby shifting adaptive stress signaling toward maladaptive inhibitory outputs (Halbleib et al., 2017; Volmer and Ron, 2015). Thus, lipid homeostasis determines whether ER stress remains within a physiological range that supports secretory adaptation or transitions into a pathological state that restricts osteogenic differentiation progression.

### 7.2 ER-mediated regulation of lipid metabolism in osteogenic differentiation

The ER functions not only as a lipid homeostasis sensing and signaling integration platform, but also actively regulates lipid metabolic transcriptional programs and membrane network remodeling, thereby contributing to cellular homeostasis maintenance and functional output control during osteogenic differentiation. Through coordinated regulation of lipid sensing, lipid biosynthesis, and membrane structural remodeling, the ER dynamically reshapes lipid metabolic programming under high secretory demand conditions, thereby determining osteoblast adaptive capacity to secretory stress and metabolic fluctuations.

The sterol-sensing and transcriptional regulatory machinery embedded in the ER membrane represents a central regulatory hub for cellular lipid homeostasis. Sterol regulatory element-binding proteins (SREBPs), the master transcriptional regulators of lipid metabolism, are synthesized as ER membrane–bound precursors and sense cholesterol fluctuations through the SCAP–INSIG complex. This process controls SREBP trafficking to the Golgi apparatus, where proteolytic activation occurs, ultimately regulating the expression of genes involved in fatty acid and cholesterol biosynthesis (Nohturfft et al., 1999; Yabe et al., 2002). Experimental evidence indicates that SREBP2 overexpression promotes cholesterol accumulation and suppresses RUNX2 expression and mineralization capacity, suggesting that the SREBP2–cholesterol homeostasis axis directly modulates osteogenic differentiation phenotypes (Zhan et al., 2025). In addition, SREBP1 and its downstream lipid metabolic gene network regulate the balance between osteogenic and adipogenic differentiation of BMSCs, highlighting a critical role of ER lipid transcriptional programs in lineage commitment regulation (Wang et al., 2025).

## 8 Therapeutic strategies

### 8.1 Reducing proteotoxic misfolding burden: ER stress-targeted therapeutic interventions

In pathological contexts characterized by markedly elevated misfolded protein burden or dominant acute stress exposure, ER stress-targeted therapeutic strategies can restore ER homeostasis and improve bone metabolic function. These strategies are better suited to pathological contexts characterized by acute increases in protein-folding demand or pathological ER stress. In contrast, under adaptive stress conditions, interventions should be approached with caution to avoid disrupting physiological secretory adaptation. ER stress–modulating strategies exhibit stage-specific features across different phases of osteogenic differentiation and pathological contexts. During early osteogenesis or acute stress conditions, maintaining moderate UPR activity supports the establishment of the secretory apparatus. In contrast, under chronic stress or high secretory load, alleviation of ER stress facilitates the maintenance of cellular function and bone matrix formation. Accordingly, therapeutic interventions should be tailored based on differentiation stage, stress intensity, and bone remodeling status. Accumulating evidence indicates that multiple ER stress modulators ameliorate bone metabolic dysfunction by maintaining adaptive UPR signaling or enhancing protein folding capacity, ER homeostasis-targeted therapeutic strategies are summarized in Table 1.

**TABLE 1:** Potential osteogenic intervention strategies targeting endoplasmic reticulum homeostasis.

Category	Intervention	Primary ER stress–related mechanisms	Mechanism of action	Evidence/Model	Suggested intervention window	Potential risks/Limitations	References
ER stress attenuation (folding-load reduction)	4-PBA	ER stress attenuation (chemical chaperone)	Enhances ER folding capacity and restrains excessive UPR activation	Disuse OP; inflammatory OP; GC-induced OP; AGEs-induced osteoblast injury	Early maladaptive ER-stress phase	Limited specificity for UPR pathways and molecular targets	Al-Dagh et al. (2024), Lee et al. (2014), Yang et al. (2017), Suzuki et al. (2020)
	Salubrinal	eIF2α signaling (ISR modulation)	Maintains adaptive ISR and osteogenic function	OVX OP; aging BMSCs dysfunction; osteoclast model	Early–mid adaptive phase	Limited UPR branch specificity; insufficient causal linkage to bone remodeling	He et al. (2013), Li j. et al. (2019), Li et al. (2022)
	Salubrinal; Guanabenz	eIF2α phosphorylation (ISR modulation)	Maintains p-eIF2α, reduces GC-induced ER stress	GC-induced OP model	Early GC stress phase	Unclear PERK specificity of eIF2α modulation; limited linkage to osteogenic outcomes	Sato et al. (2015)
	TUDCA	ER proteostasis (chemical chaperone)	Reduces ER stress and improves bone phenotype	OVX OP model	Chronic ER-stress phase	Lack of UPR pathway specificity; unclear linkage to osteogenic mechanisms	Ahn et al. (2020)
	2-APB	ER Ca^2+^ homeostasis	Reduces Ca^2+^-induced ER stress	GC-induced OP model	Early Ca^2+^ overload stage	Undefined UPR pathway involvement; limited causality of CHOP/Ca^2+^ changes	Yang et al. (2017), Guo Y. et al. (2021)
	Folic acid	ER stress–CHOP signaling	Reduces homocysteine-induced ER stress	Hyperhomocysteinemia model	Metabolic stress stage	Limited validation of PERK involvement; weak linkage to osteogenic regulation	Su et al. (2022)
	Geniposide	ER stress modulation (NRF2/GLP-1R-associated)	Reduces ER stress and apoptosis	DEX-induced OP; lipotoxic model	Early GC/metabolic stage	Unclear UPR pathway involvement; limited linkage to upstream signaling	Xiao et al. (2022), Zhong et al. (2024)
	CCT020312	PERK–eIF2α signaling	Enhances adaptive UPR	Inflammatory OP model	Early inflammatory phase	Incomplete characterization of downstream UPR signaling; limited causal validation	Tang et al. (2021)
	ED-71	ER Ca^2+^ homeostasis (SERCA2-associated)	Restores Ca2+ homeostasis	OVX OP; oxidative stress model	Chronic stress phase	Indirect ER stress regulation; limited UPR pathway characterization	Zhang Y. et al. (2025)
ER quality control enhancement (ER proteostasis/ER-phagy)	HA15	GRP78-mediated ER stress regulation	Rebalances BMSC differentiation	OVX OP; BMSC model	Early differentiation stage	Limited UPR specificity; unclear downstream signaling mechanisms	Han et al. (2021)
	BIX	BiP/GRP78-mediated ER proteostasis	Enhances ER folding capacity	Osteogenic cell model	Early differentiation stage	Insufficient characterization of UPR signaling and mechanistic specificity	Hino et al. (2010)
	Rapamycin	Autophagy/ER-phagy activation	EnhanceER quality control	hBMSCs; animal OP model	Mid-late stage	Dose-dependent effects; limited linkage to ER stress–UPR signaling	Wan et al. (2017), Xing et al. (2023)
	ERAD enhancement	Sigma-1 receptor activation	Enhances Hrd1/Sel1L-dependent ERAD activity	OVX OP; osteoclast differentiation model	High bone-turnover stage/osteoclast overactivation context	lack of osteoblast validation; limited bone formation assessment	Wei et al. (2022)
	Magnetic nanoparticle–HA scaffold	ER stress–autophagy coupling	Increases ER-stress vulnerability in osteoclasts by disrupting autophagy buffering, thereby indirectly favoring bone preservation	OVX OP model; osteoclast differentiation model	High bone-turnover stage/osteoclast-overactivation context rather than primary osteoblast-failure stage	Incomplete characterization of autophagy–ER stress crosstalk and UPR pathways	Zhu et al. (2022)

UPR, unfolded protein response; PERK, protein kinase R-like endoplasmic reticulum kinase; eIF2α, eukaryotic initiation factor 2 alpha; CHOP, C/EBP, homologous protein; GRP78/BiP, glucose-regulated protein 78/binding immunoglobulin protein; ER-phagy, endoplasmic reticulum–selective autophagy; ERAD, endoplasmic reticulum-associated degradation; SERCA2, sarco/endoplasmic reticulum Ca^2+^-ATPase, 2; BMSCs, bone marrow mesenchymal stem cells; OVX, ovariectomy; 4-PBA, 4-phenylbutyric acid; TUDCA, tauroursodeoxycholic acid.

Several ER stress modulators improve bone metabolic dysfunction through maintenance of adaptive UPR signaling or enhancement of protein folding capacity. Salubrinal, an inhibitor of eIF2α dephosphorylation, suppresses RANKL-induced osteoclast differentiation, improves bone mass and trabecular microarchitecture in ovariectomized models, and restores functional status in aging-associated BMSCs (He et al., 2013; Li J. et al., 2019; Li et al., 2022). In addition, Salubrinal and Guanabenz maintain eIF2α phosphorylation levels, thereby reducing ER proteotoxic burden, inhibiting glucocorticoid-induced osteoblast and osteocyte apoptosis, and exerting anti-osteoporotic effects (Sato et al., 2015). These agents enhance adaptive UPR signaling by sustaining eIF2α phosphorylation and may be better suited to early or adaptive phases of stress. However, prolonged maintenance of translational inhibition may impair protein synthesis and secretory function, and their dose–time–dependent effects remain to be defined.

In multiple osteoporosis models, the chemical chaperone 4-phenylbutyric acid (4-PBA) improves bone microstructure and mineralization by enhancing protein folding capacity and inhibiting UPR pathways, including GRP78–PERK/IRE1, while also suppressing osteoclast differentiation–related signaling (Al-Dagh et al., 2024; Lee et al., 2014). In glucocorticoid-induced and aging-related osteoporosis, 4-PBA further alleviates ER stress, improves mitochondrial function, and reduces osteoblast apoptosis (Yang et al., 2017; Suzuki et al., 2020). These findings suggest that 4-PBA may be more suitable for pathological conditions characterized by sustained protein-folding burden or chronic ER stress. However, as a systemic chemical chaperone, its broad activity across multiple tissues may lead to potential off-target effects, which should be carefully considered in translational settings.

Tauroursodeoxycholic acid (TUDCA) similarly alleviates ER stress and improves bone mass and trabecular architecture in ovariectomy-induced OP models (Ahn et al., 2020). Its mechanism of action involves enhancing protein-folding stability, indicating a protective role under conditions of chronic stress. In addition, proteostasis-targeting modulators improve osteoblast function through maintenance of adaptive UPR signaling. HA15 (HSPA5 inhibitor) and BIX (selective BiP inducer) respectively enhance protein folding capacity and promote osteogenic differentiation via ER proteostasis remodeling or molecular chaperone upregulation (Han et al., 2021; Hino et al., 2010). In certain pathological contexts, ER stress regulation is closely linked to bone remodeling coupling. In glucocorticoid-induced OP, dexamethasone activates the three major ER stress branches—ATF6, PERK, and IRE1—in a Ca^2+^ influx-dependent manner, thereby inducing osteoblast apoptosis, suppressing bone formation, and ultimately leading to bone mass loss and deterioration of bone microarchitecture. Pharmacological intervention using 2-APB or the chemical chaperone 4-PBA alleviates ER stress, improves osteoblast survival and function, and attenuates glucocorticoid-induced osteoporotic phenotypes (Yang et al., 2017; Guo Y. et al., 2021). In osteoporosis associated with hyperhomocysteinemia or advanced glycation end products (AGEs), ER stress mediated by the PERK–ATF4–CHOP and IRE1α–JNK signaling axes suppresses osteogenesis and accelerates bone loss, whereas targeted interventions can restore bone homeostasis (Suzuki et al., 2020; Su et al., 2022). Furthermore, these findings indicate that ER stress regulation not only affects osteoblast function but also contributes to osteoclast differentiation through pathways such as RANKL–NFATc1, suggesting that it exerts bidirectional regulatory effects in bone remodeling.

In ovariectomized OP rat models and osteoclast differentiation systems, hydroxyapatite scaffolds infiltrated with magnetic nanoparticles suppress compensatory protective autophagy, preventing osteoclasts from buffering proteotoxic misfolded protein burden, thereby enhancing ER stress-induced osteoclast apoptosis and alleviating OVX-induced bone loss (Zhu et al., 2022). Overall, alleviation of ER stress has been shown to attenuate bone loss, inhibit osteoclast differentiation, and enhance osteogenic function across various osteoporosis models. However, its translational application remains limited by several factors. Prolonged suppression of the UPR may interfere with physiological protein folding and secretory adaptation, while ER stress–modulating agents may exert off-target effects across multiple tissues. In addition, current evidence is largely derived from *in vitro* and animal studies, and systematic investigations are lacking regarding the optimal intervention window and dose–response relationships across different stages of osteogenesis and pathological subtypes.

### 8.2 Enhancing ER quality control capacity: precision modulation of ER-phagy as a core strategy

In osteoporosis-related chronic pathological conditions, osteoblasts function under sustained high secretory load and persistent ER homeostatic disruption, where alleviation of ER stress alone is often insufficient to maintain secretory adaptation capacity. In this context, ER quality control mechanisms, exemplified by ER-phagy, support osteogenic differentiation by selectively removing damaged ER subdomains and misfolded substrates, thereby preserving structural and functional homeostasis (Reggiori and Molinari, 2022; Yang et al., 2021; Smith and Wilkinson, 2017). Compared with stress-relieving strategies, ER-phagy modulation is better suited to conditions of chronic stress or sustained secretory demand, as it primarily restores ER turnover and quality control capacity rather than merely suppressing UPR signaling. In OP patient-derived hBMSCs and rat BMSCs, rapamycin-induced autophagy activation promotes osteogenic differentiation and increases ectopic bone volume and osteoid-like tissue formation in nude mouse ectopic ossification models. In contrast, the autophagy inhibitor 3-MA significantly attenuates these pro-osteogenic effects (Wan et al., 2017; Xing et al., 2023). During osteogenic differentiation, moderate enhancement of autophagy/ER quality control flux contributes to maintaining secretory homeostasis, particularly during mid-to-late stages as secretory demand increases. Notably, these studies primarily reflect changes in global autophagy activity, and their specific contributions to ER-phagy remain to be clearly distinguished. In ovariectomy-induced OP mouse models and osteoclast differentiation systems, activation of the Sigma-1 receptor enhances ER quality control via Hrd1/Sel1L-dependent ER-associated degradation (ERAD), enabling targeted degradation of SERCA2 and thereby suppressing osteoclastogenesis and mitigating OP progression. Pharmacological activation of Sigma-1 receptor signaling (e.g., dimemorfan) further strengthens ER proteostasis regulatory capacity, reduces osteoclast formation, and mitigates bone loss phenotypes (Wei et al., 2022). These findings suggest that ER quality control pathways (including ERAD and ER-phagy) not only regulate osteoblast function during bone remodeling but also contribute to osteoclast differentiation, indicating bidirectional regulatory effects in bone remodeling coupling. Notably, autophagy activators such as rapamycin exert broad effects across multiple tissues via the mTOR pathway, potentially affecting cellular metabolism and immune function. Therefore, their systemic effects and potential off-target impacts require careful evaluation in translational settings. Collectively, current evidence indicates that under persistent chronic secretory stress, enhancement of ER-phagy-mediated ER renewal and substrate clearance capacity may improve osteoblast proteostasis adaptability and functional resilience, ultimately supporting osteogenic lineage commitment and bone regeneration outcomes. Building on these findings, the selection of ER-targeted therapeutic strategies should be stratified based on specific pathological contexts. Under conditions of acute stress or elevated protein-folding burden, ER stress–relieving interventions should be prioritized to reduce folding stress and restore cellular function. In contrast, under chronic secretory demand or impaired ER structural turnover, enhancing ER-phagy–mediated quality control is more effective in maintaining osteoblast homeostatic adaptation. For conditions associated with metabolic dysregulation or Ca^2+^ dyshomeostasis, integrated interventions targeting ER–mitochondria coupling and Ca^2+^ dynamics are required. Moreover, ER-targeted strategies simultaneously modulate both osteoblast and osteoclast function during bone remodeling, and their overall effects should be evaluated in the context of bone formation–resorption coupling. Collectively, these findings support a stratified application framework for ER-targeted interventions based on stress type, differentiation stage, and bone remodeling status, thereby improving their translational potential.

## 9 Perspectives

Collectively, emerging evidence indicates that osteogenic therapeutic strategies targeting ER homeostasis are evolving from single-pathway interventions toward threshold-guided precision modulation frameworks tailored to pathological context and osteogenic stage (Wu et al., 2023). Coordinated modulation of ER stress attenuation, ER-phagy activity, and local microenvironmental conditions at defined osteogenic stages—together with consideration of dose–duration-dependent ER homeostasis regulatory features—may provide more translationally feasible therapeutic routes for OP (Song et al., 2024; Martin and Bettencourt, 2018; Cha et al., 2016; Yang et al., 2020).

Nevertheless, several key limitations remain. ER stress signaling and bone metabolic regulation form highly interconnected networks with strong dose- and time-dependent functional outputs. However, current studies still predominantly focus on single-pathway modulation or single time-point interventions, and lack systematic characterization of actionable regulatory windows across pathological contexts and osteogenic differentiation stages, thereby limiting the predictability and controllability of therapeutic outcomes (Hetz and Papa, 2018). Moreover, most current evidence is derived from *in vitro* systems or acute animal models, which may not fully capture the long-term effects of ER homeostasis modulation on osteogenic outcomes under chronic metabolic dysregulation, aging, or inflammatory conditions (Zhong et al., 2023). To address these challenges, future investigations should prioritize two key directions. First, future studies should focus on the dynamic and systems-level characterization of ER homeostasis threshold properties and stage-dependent regulatory features in osteoblasts across different pathological contexts (Hetz and Papa, 2018; Zhong et al., 2023). Second, integration of single-cell and multi-omics approaches to define cell-type-specific ER homeostasis regulatory mechanisms and the cellular heterogeneity landscape across bone-related cell populations (Li et al., 2024).

In this context, translating mechanistic insights into clinical applications represents an important direction for future research. Clinical studies examining the relationship between ER homeostasis and bone metabolism remain limited, with current evidence largely derived from small-sample clinical studies and correlational analyses, although these provide indirect support. For example, in patients with osteoporosis and metabolic bone diseases, elevated levels of ER stress–related markers (e.g., GRP78 and CHOP) and oxidative stress indicators are associated with reduced bone mineral density and imbalanced bone turnover (Zhang P. et al., 2025). In addition, preclinical and translational studies suggest that interventions targeting UPR or protein-folding burden (e.g., chemical chaperones) may have therapeutic potential in improving bone mass and microarchitecture (Al-Dagh et al., 2024). However, most existing evidence is based on cross-sectional observations or animal-derived translational inferences, and there is a lack of large-scale, longitudinal clinical studies to establish the causal role of ER homeostasis in bone metabolism and its feasibility as a therapeutic target. Therefore, future studies are warranted to integrate clinical cohort analyses with multi-omics approaches to further define the clinical utility of ER homeostasis–related biomarkers in osteoporosis risk assessment and treatment response prediction.

## 10 Conclusion

Osteogenic differentiation depends on the coordinated alignment of secretory demand and ER homeostasis rather than activation of isolated signaling pathways. By integrating protein folding capacity, Ca^2+^ homeostasis, lipid remodeling, ER stress responses, and selective ER-phagy into a unified regulatory network, the ER enables osteoblasts to meet stage-specific secretory requirements during differentiation. Framing ER regulation within a “homeostasis threshold–temporal window” paradigm provides a conceptual basis for understanding context-dependent osteogenic outcomes and highlights ER homeostasis as a promising therapeutic target for osteoporosis.
